# A Pharmacokinetic Interaction Study of Sorafenib and Iced Teas in Rats Using UPLC-MS/MS: An Illustration of Beverage-Drug Interaction

**DOI:** 10.1155/2019/2410845

**Published:** 2019-11-28

**Authors:** Hadir M. Maher, Aliyah Almomen, Nourah Z. Alzoman, Shereen M. Shehata, Amal Al-Subaie

**Affiliations:** ^1^College of Pharmacy, Department of Pharmaceutical Chemistry, King Saud University, Riyadh 11495, P.O. Box 22452, Saudi Arabia; ^2^Faculty of Pharmacy, Department of Pharmaceutical Analytical Chemistry, University of Alexandria, El-Messalah, Alexandria 21521, Egypt; ^3^Saudi Food and Drug Authority, Biologics and Evaluation Department, Riyadh, Saudi Arabia

## Abstract

Iced teas (ITs), also known as ready-to-drink teas, have gained much popularity among many nations. The modulatory effect of tea beverages on CYP3A4 increases the possibility of their potential interactions with many coadministered medications. Being a substrate of CYP3A4, sorafenib (SOR), the first-line therapy for the treatment of hepatocellular carcinoma, shows a great probability to exhibit pharmacokinetic (PK) interaction with ITs. For this purpose, different groups of Wistar rats were given oral doses of SOR (40 mg/kg), along with different types of ITs. The concentration of SOR in rat plasma was determined using UPLC-MS/MS. Chromatographic analysis was performed on a C18 analytical column, Acquity UPLC BEH™ (100 × 1.0 mm, i.d., 1.7 *μ*m particle size), using erlotinib (ERL) as an internal standard. Isocratic elution was performed with a mobile phase consisting of two solvents: solvent A (water with 0.1% formic acid) and solvent B (acetonitrile with 0.1% formic acid), in a ratio of 30 : 70, v/v, respectively. Quantitation was performed using MRM of the transitions from protonated precursor ions [M+H]^+^ to product ions at *m/z* 465.12 > 252.02 (SOR) and *m/z* 394.29 > 278.19 (ERL). The method was fully validated as per the FDA guidance for bioanalytical method validation in the concentration range of 2.5–500 ng/mL. Different PK parameters were calculated for SOR in all rat groups and groups administered with ITs and SOR, compared with groups with simply water and SOR. Experimental data revealed that ITs caused a general reduction in SOR bioavailability; an approximate reduction of 30% was recorded for all types of tested ITs. These data indicate that ITs could affect the PK profile of SOR in rats.

## 1. Introduction

Cytochrome P450 enzymes (CYP 450) are the most important drug-metabolizing enzymes (DMEs) which are responsible for the detoxification of most drug substances. Not only drugs but also daily consumed food products and beverages can affect the enzymatic activity by either inducing or inhibiting CYPs. A plethora of studies have focused on discussing the issue of drug-drug interactions between different therapeutic agents and to a lesser extent between food/beverage products and coadministered drugs [1–3].

Worldwide, tea is considered the most popular consumed beverage which comes second to water. Green tea (GT), black tea (BT), and white tea (WT) are all obtained from *Camellia sinensis* plant. They only differ in the manufacturing process and hence in their content of flavonoids known as catechins [3] which are known for their anticancer effect [4, 5]. Iced teas (ITs), also known as ready-to-drink teas, have gained much popularity among many nations. They are nonalcoholic tea beverages containing different types of tea (GT, RT, and WT), fruit flavors (e.g., apricot, peach, lemon, and strawberry), and additives (e.g., stabilizers, synthetic flavoring agents, and sweetening agents).

The modulatory effect of tea beverages on DMEs was previously studied. One study suggested that a reduction in CYP3A and CYP2C activity along with an induction of CYP1A was noticed with GT and BT [1]. Another study was concerned with the effect of RT on the regulation of CYP1A and CYP3A activity. The effect of GT polyphenols on the expression of hepatic CYP3A was shown to be dose-dependent. It was revealed that a decrease in the CYP3A activity was only noticed with high doses of GT polyphenols while the effect of low doses was nearly negligible [6]. Therefore, the modulation of CYP gene expression by tea beverages varies significantly depending on the administered dose, duration of ingestion, and the type of a particular commercial tea beverage. Moreover, the inhibitory effect of GT polyphenols on the P-glycoprotein (p-gp) transporters was also reported [7]. Thus, the possibilities of tea beverages interacting with CYP/p-gp substrates are of therapeutic importance and are of high significance. Among the reported tea-drug interactions are the tea-induced increase in the bioavailability of tamoxifen [8], simvastatin [9], and 5-FU [10].

Hepatocellular carcinoma (HCC) is among the most widely occurring cancers with high incidence of morbidity and mortality. Sorafenib (SOR) has been recognized as the first-line therapy for the treatment of HCC [11, 12] as well as different types of cancer [13–17]. SOR is a multikinase inhibitor acting by targeting both cell surface tyrosine kinase, as tyrosine kinase inhibitor (TKI), as well as an intracellular serine/threonine kinases [11, 12, 18]. SOR is primarily metabolized by CYP3A4-mediated oxidation in the liver and intestinal mucosa with p-gp-mediated active transport [19]. A literature review revealed the presence of conflicting data on the pharmacokinetics (PK) of SOR and that a large variability in intraindividual and interindividual PK is likely to occur [20]. Consequently, clinical efficacy and toxic profiles show great variability. Thus, TDM with SOR is greatly recommended in oncology practice [20–23]. It has been demonstrated that SOR shows dose-dependent adverse effects and that exposure-related toxicities are highly encountered. Since some of the patients with HCC show some degree of liver impairment, this could significantly lead to an altered SOR metabolism [21–23]. Some clinically relevant CYP3A4-mediated drug/herb-drug interactions have been reported with SOR either through CYP3A4 induction (e.g., prednisolone [24]) or inhibition (e.g., felodipine [25] and triptolide [26]). PK interaction between SOR and other coadministered kinase inhibitors (e.g., imatinib [27], erlotinib [28], lapatinib [29], and palbociclib [30]) has been previously studied. Thus, measuring SOR plasma levels is extremely important for achieving the required therapeutic outcomes particularly in patients with liver impairment or those who have experienced drug interactions [23].

Several LC-MS/MS methods have been reported for the determination of SOR in plasma samples [27–33]. Previously, we studied the effect of GT extract on the bioavailability of erlotinib and lapatinib, as examples of TKIs [34]. It was concluded that a significant reduction in plasma levels of both drugs was recorded with short-term administration compared with long-term administration. In spite of being CYP3A4 and p-gp substrates, GT caused a decrease in the bioavailability of both erlotinib and lapatinib, and a similar effect was previously reported with sunitinib [35]. It was assumed that GT caused reduction in TKIs absorption with a consequent reduction in the drug bioavailability. However, the situation with ITs seems to be more complicated because of their complex combination with different tea types of fruit/herb flavors as well as other excipients. The possible effect of each ingredient on the PK of TKIs adds to the complexity of predicted PK.

Although GT catechins' interaction with CYP/p-gp substrates has gained much interest with the rapidly increasing consumption of ITs all over the world, to our knowledge, no reports have been found so far for the interaction between ITs and SOR. Thus, this work aims to study the possible effect of ITs on PK parameters of SOR *in vivo* as a crucial beverage/drug interaction of great importance in oncology practice. In this respect, a UPLC-MS/MS method was developed and validated to measure different SOR PK parameters in rat plasma after the administration of ITs.

## 2. Materials and Methods

### 2.1. Chemicals and Reagents

**Figure 1:**
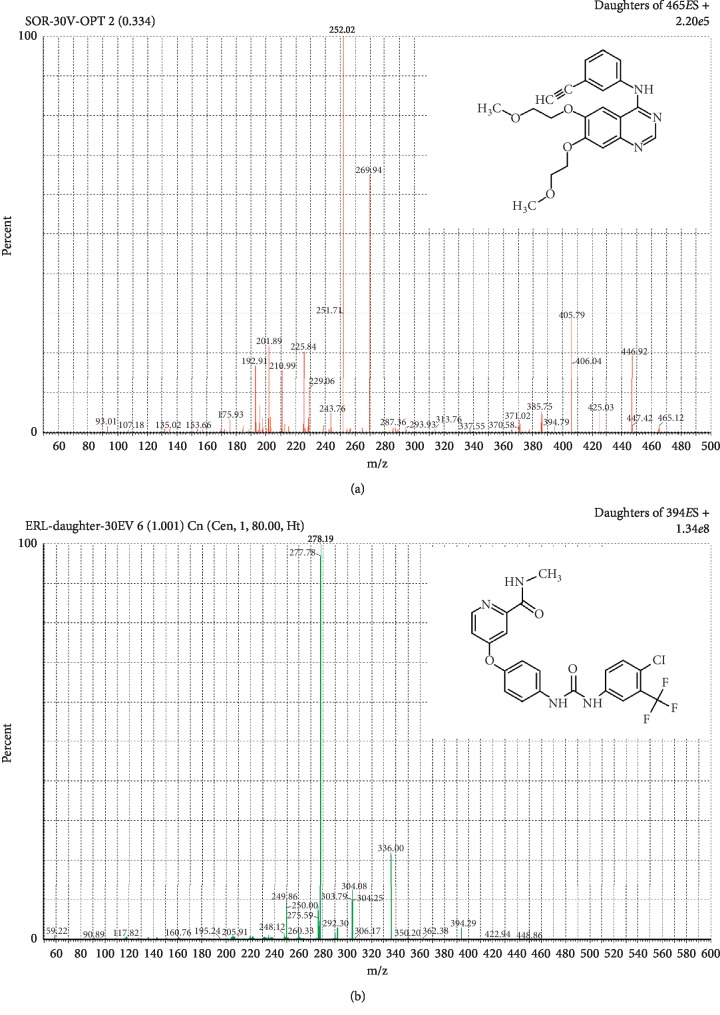
Product ion spectra of SOR (a) and ERL (b).

Sorafinib (SOR), purity ˃99%, and the internal standard (IS) erlotinib (ERL), purity ˃ 99% (Figure 1), were supplied by Haoyuan Chemexpress Co., Ltd. (Shanghai, PR China). Acetonitrile and methanol were obtained from Panreac, EU. Formic acid was purchased from Sigma Aldrich, Chemie GmbH (Steinheim, Germany). Brands, distributors, and ingredients of ITs used in this study are shown in [Supplementary-material supplementary-material-1]. Ultrapure water was produced by Milli-Q Advantage water purification system supplied with 0.22 *μ*m filters (Millipore, Molsheim, France). Different batches of rat plasma samples used for the preparation of spiked standards and quality control samples were supplied by Women Student-Medical studies and Sciences Sections, College of Pharmacy, King Saud University, Riyadh, Saudi Arabia.

### 2.2. Instrumentation and UPLC-MS/MS Conditions

Sample analysis was performed using a UPLC-MS/MS ultraperformance LC system (Waters, Singapore) supplied with a triple quadrupole mass spectrometric detector (Waters Model Xevo TQ-S) and operating with electrospray ionization (ESI) (Zspray™ ESI-APCI-ESCI) and in the multiple reaction monitoring (MRM) mode. The instrument was also connected to binary solvent manager and sample manager (Acquity™). The system was operated and controlled by Masslynx™ Version 4.1 software (Micromass, Manchester, UK). Before being introduced into the LC-MS/MS system, all samples were filtered using disposable 20/25 polyamide syringe filters (CHROMAFIL® Xtra PA) with pore size of 0.2 *μ*m and filter-Ɵ of 25 mm obtained from MACHEREY NAGEL GmbH & Co. KG (Duren, Germany).

Solid-phase extraction (SPE) cartridges operated on a JT. The Bakers vacuum system was used for sample preparation. SPE Phenomenex Cartridges Strata® C 18-E (55 *μ*m, 70 Å) (200 mg/3 mL) tubes (Torrance, USA) and Spe-ed Cartridges Octyl C 8 (200 mg, 3 mL) (Applied Separations, Allentown, Pennsylvania, USA) were involved in the study. Samples were evaporated under nitrogen using a nitrogen evaporator system N-EVAP 112 equipped with heating facility OA-SYS (Organomation Associates, Inc, Berlin, Massachusetts, USA).

The MS/MS system was operated at the positive ESI and using MRM mode for quantitation. MRM of the transitions from protonated precursor ions [M+H]^+^ to certain product ions was selected at *m/z* 465.12 > 252.02 (SOR) and *m/z* 394.29 > 278.19 (ERL). The MS conditions were adjusted as follows: source temperature 150°C, desolvation temperature 200°C, collision energy 33 eV (SOR) and 37 eV (ERL), capillary voltage 3.8 KV (SOR) and 3.5 KV (ERL), cone voltage 49 V (SOR) and 25 V (ERL), and dwell time of 0.025 s. In addition, gas flow rates were adjusted at 150 L/h, 800 L/h, and 0.15 mL/min for cone gas, desolvation gas, and collision gas, respectively. MS analyzers were operated at LM and HM resolutions of 2.8 and 14.86, respectively.

Chromatographic analysis was performed at a flow rate of 0.2 mL/min and using C18 analytical column, Acquity UPLC BEH™ (100 × 1.0 mm, i.d., 1.7 *μ*m particle size) (Waters, Dublin, Ireland). Isocratic elution was performed with a mobile phase consisting of two solvents: solvent A (water with 0.1% formic acid) and solvent B (acetonitrile with 0.1% formic acid) in the ratio of 30 : 70, v/v, respectively. Samples were injected with the partial loop with the needle overfill mode and using 5 *μ*L as the injection volume. The column temperature was maintained at 45°C while the autosampler temperature was kept at 10°C all over the run.

### 2.3. Preparation of Stock and Standard Solutions

Separate stock solutions of SOR and ERL (IS) were prepared in methanol to give final concentration of 1 mg/mL. SOR stock solution was further diluted in methanol to prepare a series of standard solutions of suitable concentrations (12.5–2500 ng/mL). For ERL, a diluted standard solution of 50 ng/mL was prepared.

#### 2.3.1. Preparation of Calibration Standards and Quality Control Samples

Calibration standards were prepared by spiking separate volumes of 50 *μ*L blank plasma samples with 100 *μ*L of the corresponding SOR standard solution (12.5, 50, 150, 300, 500, 750, 1500, 2500 ng/mL) to yield eight different concentrations of 2.5, 10, 30, 60, 100, 150, 300, and 500 ng/mL plasma of SOR. Following the addition of a constant volume of 50 *μ*L of ERL (IS) (50 ng/mL), all samples were diluted with methanol to final volumes of 1 mL. Similarly, quality control (QC) samples were prepared to give final SOR concentrations of 2.5, 7.5, 200, and 450 ng/mL for very low (LLOQ), low, medium, and high concentrations, respectively. Blank samples were prepared just by diluting 50 *μ*L plasma samples to 1 mL with methanol.

### 2.4. Sample Preparation

All plasma samples (blank or spiked) were vortexed in a micro test tube at 6000 rpm for 5 min at 4°C. From each sample, the clear supernatant was separated and the residue was further extracted with additional 0.5 mL methanol and then vortex-mixed as above. The methanolic combined supernatants were then passed on Strata® C 18-E SPE cartridges which were previously preconditioned with 3.0 mL methanol followed by 3.0 mL ultrapure water. For the purpose of elution, volumes of 0.5 mL methanol were used. The eluted methanolic solutions were evaporated to dryness using the nitrogen evaporator, and the obtained residue was then reconstituted in acetonitrile (0.5 mL). Volumes of 5 *μ*L of reconstituted samples were injected into the UPLC-MS/MS system.

### 2.5. Assay Validation

With reference to the FDA guidance for bioanalytical method validation [36], different parameters were validated.

#### 2.5.1. Specificity

Method specificity was evaluated by the analysis of blank plasma samples collected from six different batches. The resulting chromatograms were compared with those spiked with very low concentration of SOR (LLOQ, 2.5 ng/mL). The IS (ERL) was added to all samples. To test for absence of interference, the response signals at the retention times of SOR and of the IS (ERL) were recorded for both spiked and blank plasma samples.

#### 2.5.2. Linearity

Linearity was assessed using a series of plasma samples (50 *μ*L) spiked with SOR at eight different concentration levels in the range 2.5–500 ng/mL, with the IS, 50 *μ*L of 50 ng/mL ERL. Peak area ratios of SOR to ERL (IS) were related to that of SOR (IS) to construct the matrix-based calibration graph and to derive the regression equation for SOR determination.

#### 2.5.3. Lower Limit of Detection (LLOD) and Lower Limit of Quantification (LLOQ)

The lowest drug concentrations that could be detected (LLOD) or quantified with acceptable accuracy and precision of at least 20% (LLOQ) were measured. Moreover, practical evaluation were based on those analytical responses should be of at least three times (LLOD) or five times (LLOQ), compared with the blank signal measured at the same retention time of the analytes.

#### 2.5.4. Extraction Recovery

The extraction recoveries of SOR from plasma samples were evaluated at four QC concentration levels: very low LLOQ (2.5 ng/mL), low (7.5 ng/mL), medium (200 ng/mL), and high (450 ng/mL), at six replicates. Also, the recovery of ERL (IS) was assessed at its concentration level used in actual analysis. This was achieved by relating the peak response obtained from QC samples where SOR/ERL was spiked before extraction to that obtained from plasma samples spiked after extraction.

#### 2.5.5. Matrix Effect

The matrix effect of each of SOR and ERL (IS) was assessed at the same concentration levels used for assessing the extraction recovery. Recovery calculations were based on comparing the peak response from postextracted samples with that of standard solutions prepared in acetonitrile and having the same nominal concentrations.

#### 2.5.6. Precision and Accuracy

Evaluation of intraday and interday precision and accuracy was performed. QC samples (2.5, 7.5, 200, 450 ng/mL) were analyzed six times either on the same day for intraday assay or on three successive days for interday assay. In each case, the concentration of SOR was calculated by relating the obtained response (the peak area ratio of SOR to ERL (IS)) to that of freshly prepared calibration standards. The percentage relative error (*E*_r_%) and percentage relative standard deviation (%RSD) of the results were used for the assessment of accuracy and precision, respectively.

#### 2.5.7. Dilution Integrity

Plasma samples with high drug concentrations usually need dilution before actual analysis. Dilution integrity was evaluated by calculating the percentage recovery obtained from plasma samples spiked with high concentrations of SOR, beyond the linearity range (600 ng/mL). Analysis of concentrated samples was achieved following (1 : 2) and (1 : 5) dilution.

#### 2.5.8. Stability Studies

The stability of SOR in different storage and processing conditions was evaluated, in terms of percentage recoveries, by analyzing QC samples prepared at two concentration levels, low (7.5 ng/mL) and high (450 ng/mL), at six replicates. Freeze-thaw stability was assessed using three cycles of freezing plasma samples at −30°C and then thawing at room temperature (25°C). The postprocessing stability was evaluated by keeping the reconstituted plasma samples in the auto-injector at the temperature of 10°C for 56 h prior to the injection. To evaluate short-term (bench top) stability, samples were kept at room temperature (25°C) for 6 h. However, for long-term stability, samples were left frozen at −30°C for 30 days before analysis.

#### 2.5.9. Carryover Effect

Plasma samples spiked with high SOR concentrations (500 ng/mL) were analyzed using the optimized procedure. Then, three blank samples were injected into the UPLC-MS/MS system, and the obtained peak areas were recorded at the retention times of SOR and the IS.

### 2.6. Application to Pharmacokinetic Studies

#### 2.6.1. Ethics Statement

All experiments were carried out in accordance with the ethical guidelines for experimental studies with animals according to the Research Ethics committee, King Saud University (Ethics Reference no. KSU-SE-19-13).

#### 2.6.2. Study Design

Wistar healthy male rats (250 ± 30 g) were procured from the animal house, Women Student-Medical studies and Sciences Sections, College of Pharmacy, King Saud University, Riyadh, Saudi Arabia. The rats were kept for seven days before the initiation of the experiment to acclimatize under standard laboratory conditions in a well-ventilated room. These conditions were 24–27°C average temperature, 40–60% average relative humidity, and 12 h day/12 h night day cycle, with free access to water/IT and diet till 12 h prior to SOR administration. The rats were randomized into seven groups (*n* = 5) as follows:

  Group I: control group with free access to water
  Group II: testing group with free access to Lipton® peach iced tea (IT 1)
  Group III: testing group with free access to Lipton® apricot iced tea (IT 2)
  Group IV: testing group with free access to Lipton® pear and peach green ice tea, GPP (IT 3)
  Group V: testing group with free access to TAZA tazoberry® raspberry flavored black tea (IT 4)
  Group VI: testing group with free access to Rauch iced tea lemon, rose hip (IT 5)
  Group VII: testing group with free access to PASHA lemon flavored ice tea with licorice root (IT 6)

All rats except the control group had free access to the corresponding IT drink instead of water for two weeks before SOR administration. Rats of all groups (I-VII) were then treated with 40 mg/kg (1 mL of SOR 10 mg/mL in 0.9% saline) of SOR through oral gavage. Three hundred microliters of blood samples were collected through the retro-orbital sinus of each rat into a series of heparinized tubes prior to SOR administration (0 time) and at different time intervals: 0.5, 1, 2, 3, 6, 8, 24, and 48 h following drug administration. Blood samples were immediately centrifuged at 4,500 rpm (30 min, 4°C) to obtain the corresponding plasma which was stored at −20°C until the day of analysis. Each plasma sample (50 *μ*L) was spiked with 50 *μ*L of ERL, IS (50 ng/mL), and the final volume was then completed to 1 mL with methanol. Samples were then processed as discussed in Section 3.2.

#### 2.6.3. PK Calculations and Statistical Analysis

SOR plasma concentrations at the corresponding withdrawal time were treated using noncompartmental analysis (NCA) with PKSolver Add-In Excel 2010. Different PK parameters were calculated: maximum plasma concentration (*C*_max_), time taken to reach the maximum plasma concentration (*t*_max_), half-life (*t*_1/2_), the area under the curve from 0 to the last sampling time *t* (AUC_0-t_) and from 0 to ∞ (AUC_0-∞_), the area under the first moment curve from 0 to the last sampling time *t* (AUMC_0-t_) and from 0 to ∞ (AUMC_0-∞_), and mean residence time from 0 to the last sampling time *t* (MRT_0-t_) and from 0 to ∞ (MRT_0-∞_). In addition, clearance rate (CL) and volume of distribution (*V*_d_) were also calculated. Statistical significance between each testing group (II-VII) and the control group (I) was tested using Student's *t*-test at *p*=0.05.

## 3. Results

### 3.1. Method Optimization

Initially, the mass spectrometric conditions were optimized following syringe infusion of methanolic standard solutions of SOR and ERL (IS) (1 ng/mL). ESI at the positive ionization mode was selected since it provided sufficient ionization for both SOR and ERL (IS), compared with the negative mode. Practical experimentation revealed that the protonated precursor ion [M+H]^+^ was detected at *m/z* 465.12 (SOR) and 394.29 (ERL, IS). The product ion spectra (Figure 1) of SOR/ERL showed major fragments at *m/z* 252.02 (SOR) and 278.19 (ERL). Accordingly, quantitation of SOR was performed using MRM at *m/z* 465.12 > 252.02 (SOR) and *m/z* 394.29 > 278.19 (ERL). To obtain the highest response of the protonated precursor ions, the following conditions were applied: ESI source temperature of 150°C, desolvation temperature of 200°C, flow rate of desolvation gas of 800 L/h, cone voltage of 49 V (SOR) and 25 V (ERL), and capillary voltage of 3.8 KV (SOR) and 3.5 KV (ERL). However, maximum intensity of the product ions was obtained with collision energy of 33 eV (SOR) and 37 eV (ERL).

Secondly, the chromatographic conditions were optimized. Mobile phases of different ratios of acetonitrile (40–90%) and formic acid (0.05–0.15%) were evaluated for their effect on the chromatographic response of SOR and ERL (IS). Acetonitrile percentage of 70% gave the best sharpness, symmetry, and highest SOR and ERL (IS) peaks. Additionally, mobile phases with different ratios of formic acid (0.05–0.15%) in the mobile phase were tested. The best SOR sharpness and response were obtained with 0.1 % formic acid above which a decrease in peak intensity of SOR was recoded with higher formic acid content. ERL was used as an IS in SOR analysis since it produced a comparable chromatographic behavior to SOR.

### 3.2. Sample Preparation

Two SPE cartridges were evaluated for their extraction efficiency using spiked plasma samples with SOR (50 ng/mL), namely, Strata® C 18-E (55 *μ*m, 70 Å) (200 mg/3 mL) and octyl C 8 (200 mg, 3 mL). Practical experimentation revealed that C 18 cartridges provided better SOR peak shape and response compared to C 8 cartridges. Moreover, plasma samples spiked with SOR at the QC levels 2.5, 7.5, 200, and 450 ng/mL were used to assess the extraction efficiency of the selected C 18 cartridges where excellent recovery for SOR (91.24–100.57%) was obtained.

### 3.3. Method Validation

#### 3.3.1. Specificity

**Figure 2:**
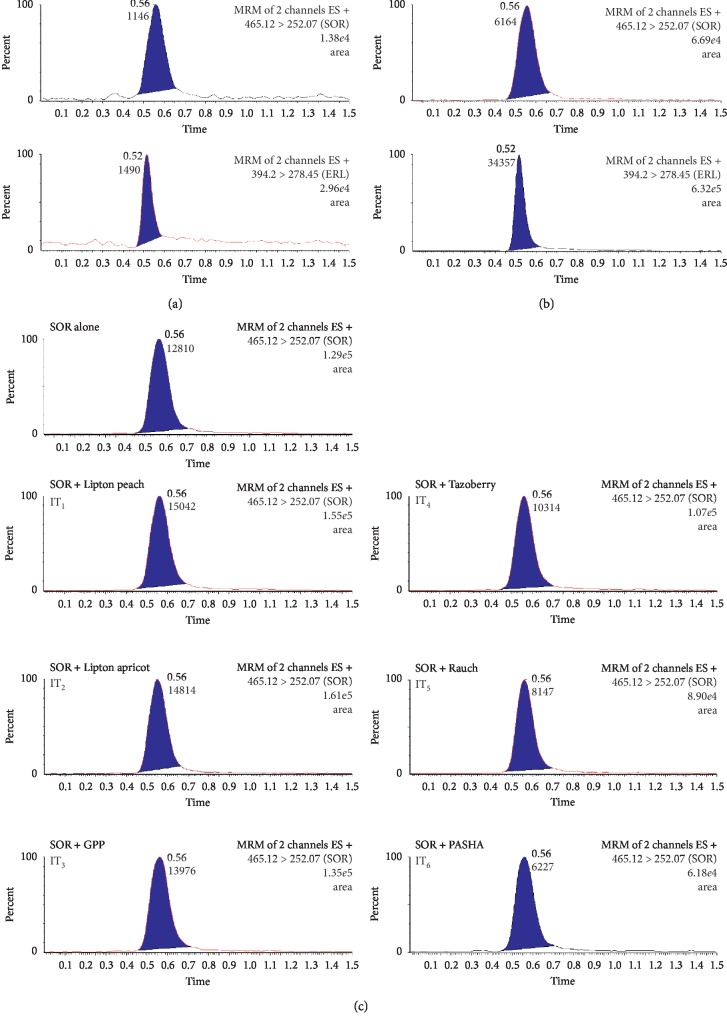
Multiple reaction monitoring (MRM) of a blank plasma (a); a plasma sample spiked with a standard mixture of SOR at its LLOQ level with ERL (IS) (b); plasma samples taken 1 h following SOR administration to rats (40 mg/kg) with either water (control group) or different types of tested ITs (c).

No interfering peaks due to endogenous components were recorded at the retention time of either SOR or ERL (IS).  Figure 2 shows typical chromatograms of blank samples and plasma samples spiked with SOR at its LLOQ level, both spiked with IS. Peak responses for SOR at its LLOQ and for the IS were at least five times and twenty times of the blank signals, respectively.

#### 3.3.2. Linearity

The method showed a good linear relationship for SOR in the range 2.5–500 ng/mL in plasma samples. The calibration curve obtained for SOR was *y* = 0.0029 + 0.0134*x*, where *y* is the peak area ratios calculated for SOR to that of ERL (IS) and *x* is the spiked concentrations, with high correlation coefficient, *r* = 0.9997. Other statistical parameters were calculated, e.g., standard deviations of residuals (*S*_*y*/*x*_) = 0.0432, of the intercept (*S*_a_) = 0.0201, and of the slope (*S*_b_) = 0.0001 and the variance ratio (*F* values) = 19284.78.

#### 3.3.3. Lower Limit of Detection (LLOD) and Lower Limit of Quantification (LLOQ)

Based on the criteria mentioned under the experimental section, SOR LLOQ and LLOD in plasma samples were 2.5 and 1.5 ng/mL, respectively.

#### 3.3.4. Extraction Recovery

The extraction recovery for SOR from plasma samples was assessed using the four QC samples, and the obtained recovery values of at least 91.24% were obtained (Table 1). Also, the recovery of ERL (IS) from spiked samples was 96.2%.

#### 3.3.5. Matrix Effect

Table 1 shows the mean values of the matrix effect at the four QC levels for SOR. Errors of less than 7.55% were obtained for SOR, while for ERL (IS), matrix effect of 7.01% was obtained.

#### 3.3.6. Precision and Accuracy

The results obtained from the analysis of the four QC samples used for assessing accuracy and precision of SOR were summarized in Table 2. The accuracy, expressed as the relative errors, were in the range (−5.14 to (−1.21) %) and (−6.59 to (−0.90) %) for intraday and interday levels, respectively. Also, method precision, calculated as RSD, fell in the range (0.27–4.43%) and (0.51–6.11%) for intraday and interday levels, respectively.

#### 3.3.7. Dilution Integrity

Table 3 revealed that recovery results (±RSD) were less than 15%.

#### 3.3.8. Stability Studies

Under all studied testing conditions, recovery values of at least 93.57% with RSD of less than 3.93 were obtained (Table 4). Also, SOR stock solutions were stable when kept refrigerated at 4°C for 3 months or kept at room temperature for 6 h.

#### 3.3.9. Carryover Effect

Blank samples which had been injected directly following the highly concentrated plasma samples showed peaks, at the retention time of SOR, with peak areas less than 20% of those produced by SOR at its LLOQ level. Also, the peaks in the chromatogram of blank plasma samples showed areas lower than 5% of the IS.

### 3.4. Application to Pharmacokinetic Studies

**Figure 3:**
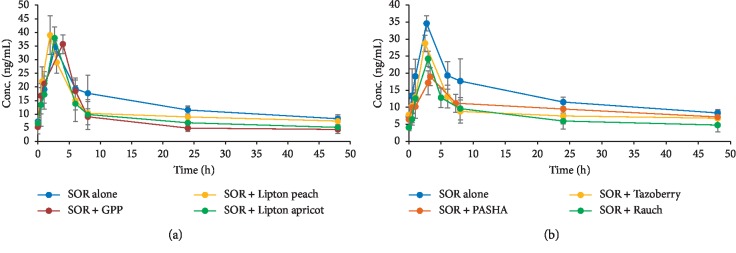
Plasma concentration-time profile of SOR in rats following oral administration of SOR (40 mg/kg) along with different types of ITs.

In this work, six types of ITs were investigated for their PK interaction with SOR. Accordingly, the study design comprised seven groups of rats (*n* = 5), six of which were given the particular IT (1 to 6) along with SOR (40 mg/kg), while the first group was used as the control group where rats were administered water instead of ITs and considering *p* < 0.05 as significant. The SOR MRM chromatograms for plasma samples 1 h following SOR administration in the seven animal groups are shown in Figure 2.  Figure 3 shows SOR plasma concentration as a function of sampling time following SOR administration: plasma concentration-time curve. Different PK parameters were calculated (Table 5). Experimental data revealed that ITs caused a significant reduction in SOR bioavailability; an approximate reduction of up to 30% in AUC_0-t_ was recorded for ITs 1 to 6. In spite of the insignificant effect on SOR *C*_max_ seen with ITs 1 to 4, a reduction of 30% and 43% was found with ITs 5 and 6, respectively. It was also noted that the administration of ITs resulted in a significant reduction in AUC_0-∞_ (IT 2, 3, 5) and/or AUMC_0-t_ (IT 2, 3, 4, 5). Moreover, increased CL was significantly found with IT 2, 3, and 5. Neither *t*_max_ nor *V*_d_ was significantly altered with any IT type.

## 4. Discussion

### 4.1. Method Optimization

To achieve maximum specificity and sensitivity, UPLC-MS/MS conditions were optimized. Different parameters affecting the chromatographic and mass spectrometric conditions were individually studied as mentioned under the “Results” section. Accordingly, the final optimized mobile phase consisted of 70% acetonitrile and 30% water, each with 0.1% formic acid, for runtime of 1.5 min. Under the abovementioned conditions, SOR was eluted at 0.56 ± 0.002 min and ERL (IS) at 0.52 ± 0.001 min, as sharp well-defined peaks.

### 4.2. Sample Preparation

The bioanalytical technique's performance relies mainly on the efficiency of sample cleanup. Different sample preparation techniques are found in the literature for the purpose of eliminating endogenous interfering compounds, e.g., protein precipitation (PPT), liquid-liquid extraction (LLE), and solid-phase extraction (SPE), with the latter being superior in the bioanalytical field particularly with LC-MS/MS. However, the great potential of combining two consecutive clean-up techniques, PPT followed by SPE, in sample purification has been previously evaluated by our research team [34, 37–41]. Thus, this combinatorial clean-up procedure was applied in this work. PPT of plasma samples with methanol was followed by further purification with SPE cartridges, Strata® C 18-E (55 *μ*m, 70 Å) (200 mg/3 mL).

### 4.3. Method Validation

#### 4.3.1. Specificity

The absence of interfering peaks at the retention time of either SOR or ERL (IS) indicated a high degree of method specificity. Moreover, peak responses for SOR at its LLOQ and for the IS (Figure 2) were at least five times and twenty times of the blank signals, respectively.

#### 4.3.2. Linearity

The calculated regression and statistical parameters and the high correlation coefficient, *r* = 0.9997, indicated high degree of linearity of the proposed method. The low value of *S*_*y*/*x*_ and the high *F* value indicated the closeness of the experimental points to the calculated regression line [42].

#### 4.3.3. Lower Limit of Detection (LLOD) and Lower Limit of Quantification (LLOQ)

The obtained SOR LLOQ shown in Figure 2 was lower than those reported in previous works [27–29, 31–33]. This ensures the applicability of the proposed method for the analysis of trace SOR concentrations in actual practice.

#### 4.3.4. Extraction Recovery

The high values of the extraction recovery obtained for both SOR and ERL (IS) from plasma samples (Table 1) indicated the efficiency of the applied method for the extraction of SOR from plasma samples.

#### 4.3.5. Matrix Effect

Evaluation of the matrix effect (Table 1) proved that the matrix-induced ion suppression or ion enhancement of the proposed method was nearly negligible with a possibility to determine very low drug concentrations in plasma samples.

#### 4.3.6. Precision and Accuracy

Obtained values for both relative errors and deviations (Table 2) were within the permitted limit, ±20.0% for LLOQ and ±15% for higher concentrations. Thus, the proposed method was considered accurate and precise at both intraday and interday levels.

#### 4.3.7. Dilution Integrity

Recovery results (±RSD) of less than 15%, as specified by the FDA guidance, indicated the integrity of SOR in concentrated plasma samples up to five times dilution.

#### 4.3.8. Stability Studies

The stability of SOR was revealed from recovery values (RSD) which did not exceed the acceptance limit of ±15%.

#### 4.3.9. Carryover Effect

The obtained results showed that the carryover effect was negligible for the analysis of SOR by the proposed UPLC-MS/MS method.

### 4.4. Application to Pharmacokinetic Studies

During the last decades, ITs gained much popularity among different nations, being served as canned or bottled tea beverages. Manufacturers usually serve ITs as packaged drinks of herbal teas, flavored syrup, sweeteners, and preservatives. The widespread consumption of ITs, along with its complex nature, has contributed to the possible increase in PK interactions with coadministered therapeutic agents. Drug interactions with TKIs have recently gained much attention as a limiting factor in the efficiency of drug protocols. This attracts our attention to study beverage/drug interaction with TKIs. An example of which is the interaction of ITs with SOR, the first-line TKI used in the treatment of HCC. Although, like other TKIs, SOR is a substrate for both CYP3A4 and p-gp and that ITs have inhibitory effect on CYP3A4 and p-gp, but its bioavailability is decreased by the coadministration of ITs. This unexpected finding was previously reported with GT administration with other TKIs: erlotinib, lapatinib [34], and sunitinib [35]. This could be attributed to a reduction in the fraction of the absorbed drug in the stomach as a result of tea beverages coadministration. Hence, further studies are needed to better understand the exact mechanism of IT-induced reduction in SOR bioavailability. The complex nature of ITs also suggests the necessity of studying the effects of the individual components on modulating DMEs and/or transporters. Also, this work should be extended to clinics to confirm the possibility of IT effect on SOR PKs in human subjects. Finally, care should be paid with the intake of ITs while on SOR therapy, which may result in therapeutic failure and/or acquired resistance. This supports the ultimate need for TDM in patients administered TKIs in oncology practice.

### 4.5. Advantages of the Proposed Method over Previously Published Methods

Compared with previously published LC-MS/MS bioanalytical methods [27–33], the proposed method provided higher detectability and lower LLOQ, for the determination of SOR in plasma. This is extremely important in trace determination of SOR in plasma samples, an important issue in terminal phase elimination. The obtained low values of LLOQ for SOR can be attributed to the combinatorial sample preparation technique since the use of protein precipitation followed by SPE, as compared with merely protein precipitation in previous work, provided potential cleanup of plasma samples with high degree of elimination of interfering plasma components. Moreover, high throughput of the proposed method was maintained by the short runtime, only 1.5 min was required for the whole run, compared with relatively longer times in previous methods [27–33].

## 5. Conclusion

A simple, selective, and fast UPLC-MS/MS method was developed and validated to study the effect of ITs on the PK parameters of SOR in Wistar rats. This is crucial since ITs are reported to have a modulatory effect on CYP 450, the main metabolizing enzyme of TKIs including SOR. Practical experimentation revealed that ITs decrease the bioavailability of SOR in rats, hence possibly reducing the efficiency of treatment and increasing the chance of an acquired resistance. Thus, the coadministration of ITs with SOR should be avoided especially that there has been an increasing demand to perform TDM with TKIs. Since this study revealed that PK interaction of ITs and SOR existed in rat, this interaction studies should be extended to the human level and the mechanism of interaction should be further evaluated.

## Figures and Tables

**Table 1:** Evaluation of the extraction recovery and matrix effect for the determination of SOR in rat plasma by the proposed UPLC-MS/MS method.

Concentration added (ng/mL)	Extraction recovery		Matrix effect	
	Mean recovery (%) ±RSD	*E*_r_ (%)	Mean recovery (%) ±RSD^a^	*E*_r_ (%)
2.5	92.68 ± 3.01	−7.32	92.45 ± 4.44	−7.55
7.5	91.24 ± 2.76	−8.67	97.67 ± 0.91	−2.33
200	100.57 ± 3.32	0.57	95.67 ± 2.26	−4.33
450	98.92 ± 2.01	−1.08	99.35 ± 0.58	−0.65

Mean recovery (%) ±RSD of six determinations. *E*_r_ (%): percentage relative error.

**Table 2:** Evaluation of the intraday and interday accuracy and precision for the determination of SOR in rat plasma by the proposed UPLC-MS/MS method.

Concentration added (ng/mL)	Intraday (*n* = 6)		Interday (*n* = 18)	
	Mean recovery (%) ±RSD	*E*_r_ (%)	Mean recovery (%) ±RSD	*E*_r_ (%)
2.5	94.86 ± 4.43	−5.14	95.73 ± 6.11	−4.27
7.5	96.63 ± 3.52	−3.37	93.41 ± 1.57	−6.59
200	98.79 ± 0.27	−1.21	96.87 ± 2.81	−3.13
450	97.97 ± 2.45	−2.03	99.10 ± 0.51	−0.90

Mean recovery (%) ±RSD of six determinations. *E*_r_ (%): percentage relative error.

**Table 3:** Evaluation of the dilution integrity of SOR in rat plasma.

Concentration spiked (ng/mL)	Dilution fold	Mean recovery (%) ±RSD	*E*_r_ (%)
600	1 : 2	97.37 ± 1.67	−2.63
	1 : 5	97.10 ± 1.95	−2.90

Mean recovery (%) ±RSD of six determinations. *E*_r_ (%): percentage relative error.

**Table 4:** Evaluation of the stability of SOR in rat plasma.

Stability	Concentration added (ng/mL)	Mean recovery (%) ±RSD
Autosampler stability (10°C, 56 h)	7.5	97.64 ± 1.87
	450	98.25 ± 3.11
Short-term stability (25°C, 6 h)	7.5	98.13 ± 2.39
	450	100.47 ± 2.72
Long-term stability (−30°C, 30 days)	7.5	97.54 ± 3.93
	450	95.87 ± 2.33
Freeze-thaw stability (−30°C, 3 cycles)	7.5	93.57 ± 1.68
	450	99.05 ± 2.39
Refrigerator (4°C, 3 months)	7.5	96.66 ± 3.78
	450	98.86 ± 1.39

Mean recovery (%) ±RSD of six determinations.

**Table 5:** Main pharmacokinetic parameters (mean ± SD) after oral administration of SOR to rats (*n* = 5).

	Group I SOR (40 mg/kg)	Group II SOR (40 mg/kg) + Lipton peach (IT 1)	Group III SOR (40 mg/kg) + Lipton apricot (IT 2)	Group IV SOR (40 mg/kg) + GPP (IT 3)	Group V SOR (40 mg/kg) + TAZA tazoberry (IT 4)	Group VI SOR (40 mg/kg) + Rauch (IT 5)	Group VII SOR (40 mg/kg) + PASHA (IT 6)
*C*_max_ (ng/mL)	34.67 ± 2.21	38.93 ± 4.82	37.83 ± 4.29	37.13 ± 3.56	28.78 ± 2.45	24.35 ± 2.78^*∗*^	19.95 ± 2.29^*∗*^
*t*_max_ (h)	2.74 ± 0.99	1.95 ± 0.74	2.52 ± 0.22	3.04 ± 0.72	2.50 ± 0.57	2.98 ± 0.31	3.31 ± 0.72
*t*_½_ (h)	36.48 ± 2.46	85.34 ± 3.71^*∗*^	42.90 ± 2.73	39.76 ± 2.86	107.22 ± 6.53^*∗*^	40.67 ± 1.95	62.72 ± 3.02^*∗*^
AUC_0-t_ (ng·h/mL)	659.50 ± 45.64	487.41 ± 29.15^*∗*^	448.61 ± 24.76^*∗*^	408.43 ± 34.61^*∗*^	439.43 ± 48.17^*∗*^	384.75 ± 27.93^*∗*^	44.03 ± 42.84^*∗*^
AUC_0-∞_ (ng·h/mL)	1093.86 ± 98.64	1438.29 ± 147.24	761.61 ± 23.87^*∗*^	655.14 ± 36.28^*∗*^	1477.05 ± 221.41	644.14 ± 36.29^*∗*^	1116.53 ± 133.15
AUMC_0-t_ (ng·h/mL)	12095.06 ± 1062.69	9813.53 ± 753.59	7432.71 ± 955.17^*∗*^	6125.81 ± 755.53^*∗*^	8474.67 ± 777.36^*∗*^	6562.05 ± 543.03^*∗*^	9843.53 ± 947.78
AUMC_0-∞_ (ng·h/mL)	52579.53 ± 1853.53	165795.6 ± 25471.07^*∗*^	42731.1 ± 2039.52^*∗*^	32883.51 ± 1887.29^*∗*^	224631.6 ± 25471.07^*∗*^	36043.20 ± 2846.87^*∗*^	98498.35 ± 977.33^*∗*^
MRT_0-t_ (h)	18.67 ± 0.92	18.72 ± 1.05	16.88 ± 0.55	15.21 ± 0.94^*∗*^	18.42 ± 0.80	18.22 ± 1.06	20.61 ± 1.24
MRT_0-∞_ (h)	52.52 ± 4.44	114.43 ± 12.49^*∗*^	55.93 ± 6.26	50.13 ± 0.79	149.91 ± 24.53^*∗*^	56.46 ± 5.16	88.36 ± 8.68^*∗*^
CL (L/h/kg)	36.04 ± 4.88	27.77 ± 1.43	52.38 ± 2.80^*∗*^	60.83 ± 8.91^*∗*^	26.48 ± 3.08	63.68 ± 5.91^*∗*^	35.86 ± 4.87
*λ*_z_ (h^−1^)	0.0184 ± 0.0032	0.0083 ± 0.0007^*∗*^	0.0161 ± 0.0024	0.0176 ± 0.0052	0.0065 ± 0.0017^*∗*^	0.0176 ± 0.0071	0.0113 ± 0.0029
*V*_d_ (mL/kg)	1.96 ± 0.77	3.39 ± 0.33	3.23 ± 0.68	3.51 ± 0.78	4.18 ± 0.87	3.61 ± 1.14	3.24 ± 0.64

^*∗*^Significant difference as compared with group I (*p*=0.05).

## Data Availability

The data used to support the findings of this study are available from the corresponding author upon request.

## Supplementary Materials

Supplementary Materials Table S1: distributor and ingredients of iced tea products involved in the study.
