# Supplementary material for: A Pharmacokinetic Interaction Study of Sorafenib and Iced Teas in Rats Using UPLC-MS/MS: An Illustration of Beverage-Drug Interaction
Source: Biomed Res Int. 2019 Nov 28;2019:2410845. doi: 10.1155/2019/2410845 (PMC6907072; doi:10.1155/2019/2410845)
Supplement: Supplementary Materials — Table S1: distributor and ingredients of iced tea products involved in the study. [file 2410845.f1.docx]

Table 1 supplementary

| Iced tea | Distributor | Ingredients |
| --- | --- | --- |
| Lipton^®^ Peach  Ice tea | Britvic Soft Drinks Ltd., Hemel Hempstead, HP2 4TZ, UK. Britvic Ireland Ltd. (Dublin, Ireland). | Water, Sugar, Acids (Malic Acid, Citric Acid), Black Tea Extract (0.14%), Peach Juice from Concentrate (0.1%), Flavourings, Antioxidant (Ascorbic Acid), Acidity Regulator (Trisodium Citrate), Sweetener (Steviol Glycosides). |
| Lipton^®^ Apricot  Ice tea | Industrial Refreshments Company LTD, 2^nd^ Industrial City, Riyadh, Saudi Arabia | Water, Sugar, Acids (Citric Acid), Black Tea Extract (0.12%), Nature identical apricot flavor, Acidity Regulator (sodium Citrate), Antioxidant (Ascorbic Acid), Sweetener (Steviol Glycosides 27mg/mL).  (No colorants, no preservatives) |
| Lipton^®^ Pear & Peach  Green Ice tea | Industrial Refreshments Company LTD, 2nd Industrial City, Riyadh, Saudi Arabia | (Non-carbonated drink with tea extracts and pear and peach flavor)  Water, sugar, green tea extract (0.13%), acidulant (citric acid, malic acid), nature identical peach flavouring and nature identical pear flavouring, antioxidant (ascorbic acid), acidity regulator (sodium citrate), sweetener (steviol glycosides 80 mg/L) |
| TAZA  tazoberry^®^  black tea | TAZO, USA | A tea infusion of (water, blended black teas), cane sugar, ***apple and raspberry concentrate juices***, natural raspberry flavors, citric acid. |
| Rauch  Iced tea lemon, rose hip | Rauch Fruchtsa^..^fte, France | Infusion of black tea and rose hip (water, black tea, rose hip), sugar, 1.5% lemon juice from concentrate, acid (citric acid), acidity regulator (sodium citrate), natural lemon aroma |
| Meyania  PASHA  Ice tea | Turkey | (Lemon Flavored Ice Tea with Licorice Root)  Water, ***fresh brewed liquorice***, black tea extract, natural herbal sweeteners (stevia glycosides), antioxidant (ascorbic acid), acidity regulator (citric acid, sodium citrate), ***natural lemon flavor*** |
